# Vitamin D: The Missing Nutrient Behind the Two Deadly Pandemics, COVID-19 and Cardiovascular Diseases

**DOI:** 10.7759/cureus.24133

**Published:** 2022-04-14

**Authors:** Abhishek Singh, Anusha Chidharla, Kriti Agarwal, Priyanka Singh, Nidhi Jain, Gashaw Hassen, Salwa Abdelwahed, Renu Bhandari, Kajal Patel, Sachin Gupta, Thoyaja Koritala, Rizwan Rabbani

**Affiliations:** 1 Internal Medicine, Saint Vincent Hospital, Worcester, USA; 2 Hematology Oncology, University of Kansas Medical Center, Kansas, USA; 3 Department of Internal Medicine, Hackensack Meridian Health, Palisades Medical Center, North Bergen, USA; 4 Medicine, Smolensk State Medical University, Smolensk, RUS; 5 Cardiology, Apex Heart and Vascular Care, New Jersey, USA; 6 Medicine and Surgery, Himalayan Institute of Medical Sciences, Dehradun, IND; 7 Hematology and Oncology, Brooklyn Cancer Care, Brooklyn, USA; 8 Internal Medicine, Sir Ganga Ram Hospital, New Delhi, IND; 9 Internal Medicine, Mercy Medical Center, Baltimore, USA; 10 Department of Family and Community Medicine, University of Missouri Kansas City School of Medicine, Kansas City, USA; 11 Internal Medicine, Manipal College of Medical Sciences, Kaski, NPL; 12 Internal Medicine/Family Medicine, California Institute of Behavioral Neurosciences & Psychology, Fairfield, USA; 13 Department of Internal Medicine, Smt. Kashibai Navale Medical College and General Hospital, Nahre, IND; 14 Internal Medicine, Penn State Health Milton S. Hershey Medical Center, Hershey, USA; 15 Internal Medicine, Mayo Clinic, Mankato, USA; 16 Nephrology, Temple University Hospital, Philadelphia, USA

**Keywords:** peripheral arterial disease (pad), stroke, coronary heart disease (chd), acute myocardial infarction (ami), cytokines, icu admissions, vitamin-d deficiency, cytokine storm syndrome, vitamin-d, covid 19

## Abstract

The coronavirus (COVID-19) pandemic is claiming millions of lives and creating an additional burden on health care, which is already affected by the rise of non-communicable diseases (NCDs). The scientific community, on the other side, is enormously engaged with studies to best identify the characteristics of the virus and minimize its effect while supporting the fight to contain NCDs, mainly cardiovascular diseases (CVDs), which are contributing hugely to the global death toll. Hence, the roles of vitamin D in COVID-19 immunity and cardiovascular health are gaining traction recently.

This literature review will mainly focus on summarizing pertinent studies and scientific publications which highlight the association of vitamin D levels with the various outcomes of COVID-19 and CVDs. It will also address how low vitamin D correlates with the epidemiology of CVDs and the inflammatory mechanisms attributed to COVID-19 severity. We believe that our review may open up hindsight perspectives and further discussions among the physicians in tapping the potential of vitamin D supplementation to tackle the morbidity, mortality, and health care cost of the two deadly diseases, COVID-19 and CVDs.

## Introduction and background

Vitamin D is known for its regulation of calcium levels and for maintaining bone health [1]. However, recent studies suggest that it also has immunomodulatory and anti-inflammatory action. Vitamin D as a strong epigenetic regulator on more than 2500 genes, contributes to its influence on various diseases. Thus, it has an association with serious illnesses like cancers, diabetes mellitus (DM), acute respiratory infections (ARIs), autoimmune diseases like multiple sclerosis, and interestingly cardiovascular diseases (CVDs), the leading cause of death among the non-communicable diseases globally [2-8].

As of February 2022, the total burden of COVID-19 cases in the United States was more than 75.6 million, with mortality exceeding 0.89 million [9]. In the study using the National Health and Nutrition Examination Survey (NHANES) 2011-2012 database, 4962 participants were surveyed and examined in 1981 (39.92%) were found to be vitamin D deficient, which was in concordance with prior data collected in 2005-2006 (also by NHANES) [10]. Certain findings seen in recent studies indicate COVID-19 association with vitamin D status, such as:

Seasonal variation: showing inverse correlations with solar Ultraviolet B (UVB) doses and vitamin D production [11-12]. COVID-19 cases started in winter in the northern hemisphere, and decreased number of cases and death rates were seen in summer, especially in Europe, and increased rates were seen later in July-September in various European countries [13].

Racial disparity: as seen with higher COVID-19 cases and death rates in African Americans and Hispanics than European Americans, most likely due to lower vitamin D levels and dark skin [14-16].

While the world is grappling with the COVID-19 outbreak, the pandemic nature of CVDs occurring in parallel is discussed to a lesser extent among the scientific community. Non-communicable diseases (NCDs), mainly CVDs, are killing more people than infectious pandemics accounting for 71% of global mortality [17,18]. CVDs, including coronary heart disease and cerebrovascular disease, are the leading cause of death globally and contributed to 17.9 million deaths in 2019, an estimated 32% of all deaths worldwide [19]. Applying the definition of a pandemic in the sense of a health condition that spans and burdens globally, some literature describes CVDs as ‘the invisible pandemic’, ‘the forgotten pandemic’, ‘the impending global pandemic’, and ‘a pandemic hiding in plain sight’ [20-25].

Vitamin D is not only missing as an important nutrient from those infected by the COVID-19 but also from patients suffering from CVDs, which affect their heart and blood vessels. A recent review by Latic N and Erben RG highlighted the role of vitamin D in cardiovascular health and the association of hypertension, atherosclerosis, and heart failure with vitamin D deficiency which by itself has affected one billion world population [26]. Despite the lack of sound interventional studies which prove the causal relationship between vitamin D supplementation and its beneficial effects on cardiovascular health, most observational and ecological studies strongly support the cardiovascular protective effect of 25(OH)vitamin D [26,27].

## Review

Vitamin D deficiency and COVID-19 mortality due to cytokine storm 

Cannell et al. in 2015 revealed that vitamin D can lower the production of specific cytokines, including IL-6, in people with inflammatory medical problems [28]. A recent study by McElvaney et al. and two separate reviews by Mahmudpour et al. and Koritala et al. highlighted the presence of a “cytokine storm”, which is an out-of-control release of inflammatory cytokine as one of the hallmarks of COVID-19 infection [29-31]. 

Also, few other studies have demonstrated a higher number of COVID-19-related deaths, particularly in patients with high IL-6 cytokine levels. In the UK, males had 72% higher death rates than females, which, when compared to the study published by Wei et al., showed UK men had 112% higher levels of IL-6 than the mean female levels [32-33]. Similarly, black patients in the UK had death rates 40 percent higher than white patients, aside from the fact that IL-6 levels in black patients were 59 percent higher in the US trial [32,34]. Obese patients died at a rate of 48 percent and higher than non-obese patients in the UK, plus the fact that IL-6 levels in obese patients are 300 times higher than in non-obese patients, according to a US study. [32,35]. Although further research is needed to determine the link between vitamin D supplements, IL-6, and COVID-19 endpoints, existing research suggests that vitamin D prescription might be used for its preventative as well as therapeutic role [36].

The interplay between vitamin D and COVID-19 immunity

Vitamin D could play a vital role in reducing the risk, severity, and death from COVID-19 infection through the following possible mechanisms. Regulatory T lymphocytes (Tregs) levels have been reported to be markedly decreased in severe cases. Tregs levels can be increased by supplementing vitamin D [37-38]. Low levels of vitamin D are also associated with an increase in inflammatory cytokines. It has been shown that vitamin D decreases levels of IL-6 and TNF-ɑ [39].

Vitamin D and cardiovascular diseases

Current evidence supports the role of vitamin D in the severity/outcomes of cardiovascular diseases (CVDs), as shown in the studies below. (Table 1)

**Table 1:** Role of isolated vitamin D deficiency in CVDs

Study Author	Study Type	Sample Size	Outcomes Considered	Findings
Wang et al. [40]	Meta-analysis of 19 independent studies	6123 CVD cases among 65994 participants	Incident MI, incident stroke, total CVD, mortalities due to CVD, stroke, and coronary heart disease (CHD)	Comparing the lowest to the highest 25(OH)-vitamin D categories, the pooled RR (95% CI) was 1.52 (1.30–1.77) for total CVD, 1.42 (1.19–1.71) for CVD mortality, 1.38 (1.21–1.57) for CHD, and 1.64 (1.27–2.10) for stroke
Kim et al. [41]	A cross-sectional study on the prevalence of hypovitaminosis D in adults with CVDs using data from NHANES 2001 to 2004	8351 adults	CHD, heart failure (HF), peripheral arterial disease (PAD)	In hypovitaminosis D (<20 ng/ml), adjusted OR (for age, race and gender) was 1.49 with 95% CI [1.17,1.91] for CHD, 2.10 with 95% CI [1.24,3.56] for HF, 1.14 with 95% CI [0.76,1.72] for stroke and 1.82 with 95% CI [1.26,2.61] for PAD
Tomaschitz et al. [42]	A cohort study to determine the association between 25-hydroxyvitamin D [25(OH)D], 1,25-dihydroxy vitamin D [1,25(OH)2D], and the renin-angiotensin system (RAS) in patients referred for coronary angiography	3316 subjects	Circulating RAS levels	Age and gender-adjusted analysis of (co) variance (ANCOVA) revealed mean plasma renin concentration (PRC), angiotensin 2 levels, and plasma aldosterone concentration (PAC) values significantly higher in vitamin D deficient patients (25-hydroxyvitamin D < 20 μg/L) in comparison with sufficient vitamin D levels (25- hydroxyvitamin D>30μg/L). (P-value < 0.001 for PRC and angiotensin 2; P-value = 0.045 for PAC).
Aleksova et al. [43]	A cross-sectional study on the prevalence of vitamin D deficiency in acute myocardial infarction (AMI)	478 subjects diagnosed with AMI	AMI	Vitamin D deficiency in 324 (68 %) and insufficiency in 107 (22 %) subjects with AMI.
Lee et al. [44]	Prevalence study on subjects enrolled in Translational Research Investigating Underlying Disparities in AMI Patients’ Health Status (TRIUMPH) registry at 24 US hospitals from April 11, 2005, to December 31, 2008	239 patients	AMI	The study revealed that at baseline: 179 subjects (75%) were in the deficient range of 25(OH)D levels <20 ng/ml, another 50 subjects had vitamin D levels of 20 to 30 ng/ml, which is in the insufficient range. Resulting in a total of 229 of 239 subjects (96%) in the suboptimal range of 25(OH)D (normal range >30 ng/ml)
Belen et al. [45]	A prospective cross-sectional trial was conducted at the Hypertension Outpatient Clinic of Okmeydanı Training and Research Hospital, Istanbul, Turkey, from September 2013 to April 2014	150 subjects	Resistant Hypertension (RH)	Multivariate regression analysis showed that 25-hydroxyvitamin D levels remained the only independent correlate of RH in the study population (β 0.660, 95% CI 0.572-0.760, p < 0.001).
Sun et al. [46]	Prospective case-control study of US female registered nurses aged 30 to 55 years and meta-analysis of six other prospective cohort studies which included both men and women	464 case-control pairs and additional 1214 stroke cases were included in the meta-analysis	Stroke	Individual Nurses' health study showed the odds ratio, OR (95% CI), comparing women in the lowest vs. highest tertiles was 1.49 (1.01–2.18; P_trend_=0.04) after multivariable adjustment. The pooled relative risk of the six prospective studies included in the meta-analysis, RR (95% CI), was 1.52 (1.20–1.85; I^2^=0.0%, P_heterogeneity_=0.63)

It is obvious from the table that various studies were able to reiterate how vitamin D deficiencies and insufficiencies are highly identified in patients with cardiovascular morbidities and mortalities. Patients with low vitamin D are more likely to have higher CVDs like AMI, CHD, HF, PAD, RH, and stroke, in addition to increased circulating RAS levels. Do CVDs play some roles in the severity, morbidity, and mortality of COVID-19 in patients with hypovitaminosis-D? This important question needs further investigation by others as this review focuses more on Vitamin D's role in COVID-19. 

Vitamin D and acute respiratory infections

The existing experimental data on the effects of vitamin D supplementation on the prevention and treatment of acute respiratory infections (ARIs) in the general population is ambiguous. Furthermore, there is insufficient evidence to conclude the influence of vitamin D supplementation on the severity or length of ARIs, as well as outcomes related to lung damage or ARI-related hospitalization. Vitamin D deficiency (50 nmol/L [20 ng/mL]) and insufficiency (75 nmol/L [30 ng/mL]) are linked to an elevated risk of ARIs, and supplementation for people with deficiency/insufficiency may result in clinically relevant decreases in the frequency of ARIs. Vitamin D supplementation appears to offer a high margin of safety, according to existing experimental evidence, with very few adverse effects reported in children and adults using various dosing techniques [47]. 

Other studies are questioning the benefit of vitamin D supplementation based on contradictory and inconsistent results. A recent study found that vitamin D in high-dose intermittent bolus, which paradoxically may induce vitamin D inactivating 24-hydroxylase, and fibroblast growth factor 23 failed in preventing rickets [37]. Trials in tuberculosis (TB) and other diseases, as well as meta-analyses of vitamin D supplementation in the prevention of ARIs, indicate the effectiveness of low dosage daily maintenance rather than intermittent bolus doses. Given the well-documented links between COVID-19 risk and vitamin D insufficiency, this is especially important during the present COVID-19 pandemic. We strongly encourage physicians to take notice of these results and to promote the broad use of vitamin D supplementation regularly [48].

Vitamin D supplementation and clinical outcome of COVID-19 patients

The number of deaths during the 1918-1919 "Spanish flu" pandemic was significantly decreased when patients were treated in "open-air" hospitals with access to sunshine, possibly owing to vitamin D's inhibition of "cytokine storm" [49]. Before effective anti-tubercular therapy, effective TB therapies included cod liver oil, UVB phototherapy, and sunlight, all of which are vitamin D sources [50].

A study done by Alipio M. found that of 212 (100.0%) cases of COVID-19, 49 (23.1%) were identified as mild, 56 (26.4%) were severe, and 48 (22.6%) were critical. The mean serum 25(OH)D level was 23.8 ng/ml. Patients with serum 25(OH)D levels above the mean had mild outcomes, while those below the mean had worst outcomes showing levels of 21.2 ng/ml for severe and 17.1 ng/ml for critical patients. Serum 25(OH)D levels were statistically significant among clinical outcomes (p<0.001). In the same study, 77 (36.3%) cases were identified as vitamin D-deficient, the majority of which were severe (40.3%). In addition, with every standard deviation rise in serum 25(OH)D, the likelihood of having a mild rather than a severe clinical result increased by 7.94 times (OR=0.126, p0.001). While it's worth noting that the probabilities of having a mild clinical result rather than a catastrophic outcome were about 19.61 times higher (OR=0.051, p0.001). More generally, the odds of having a mild clinical outcome increase when serum 25(OH)D level increases [51].

Interestingly, a pilot randomized clinical trial (RCT) published in October 2020 demonstrated that administration of a high dose of Calcifediol (25(OH)D) significantly reduced the need for ICU treatment of patients requiring hospitalization due to proven COVID-19 [52].

Immunologic mechanisms behind cytokine storm in COVID-19

A study done by Soy et al. found that SARS-CoV-2 cellular entrance is dependent on S proteins on the virion's surface binding to the cellular ACE2 receptor and S protein priming by TMPRSS2, a host membrane serine protease. After infecting respiratory epithelial cells, SARS-CoV-2 triggers an immunological response that includes the generation of inflammatory cytokines and a modest interferon (IFN) response. The proinflammatory immune responses of pathogenic Th1 cells and intermediate CD14+CD16+monocytes are mediated by membrane-bound immune receptors and downstream signaling pathways. This is followed by macrophage and neutrophil infiltration into the lung tissue, resulting in a cytokine storm [53].

SARS-CoV-2 has the potential to swiftly activate pathogenic Th1 cells, prompting them to generate proinflammatory cytokines such as GM-CSF and interleukin-6 (IL-6). Furthermore, GM-CSF activates CD14+CD16+ inflammatory monocytes, prompting them to produce large levels of IL-6, TNF-, and other cytokines. Membrane-bound immune receptors (such as Fc and Toll-like receptors) may contribute to an imbalanced inflammatory response, and insufficient IFN-induction may act as a cytokine amplifier. Extracellular nets produced by neutrophils, known as neutrophil extracellular traps, may have a role in cytokine release. The elevated expression of IL-6 and TNF- ɑ in COVID-19 is indicative of a cytokine storm. Hirano and Murakami suggested the angiotensin 2 (AngII) pathway as a possible cause of the cytokine storm [54]. 

Nuclear factor-B (NF-B) is activated by SARS-CoV-2 through pattern-recognition receptors. It binds to ACE2 on the cell surface, causing a decrease in ACE2 expression and an increase in AngII. The AngII-angiotensin receptor type 1 axis may also generate TNF- ɑ and the soluble form of IL-6Ra (sIL-6Ra) through disintegrin and metalloprotease 17 in addition to activating NF-B (ADAM17). The IL-6-sIL-6R complex is formed when IL-6 binds to sIL-6R through gp130, and it may activate the signal transducer and activator of transcription 3 (STAT3) in nonimmune cells. Both NF-B and STAT3 may activate the IL-6 amplifier, resulting in the production of a variety of proinflammatory cytokines and chemokines such as vascular endothelial growth factor (VEGF), monocyte chemoattractant protein 1 (MCP-1), IL-8, and IL-6. IL-6 can not only act in cis-signaling by binding to sIL-6R, but it can also act in trans-signaling by binding to the membrane-bound IL-6 receptor (mIL-6R) through gp130. The latter may cause cytokine storms by having pleiotropic effects on acquired and innate immune cells. SARS-CoV-2 may induce cytokine storms as a result of defective acquired immune responses and uncontrolled inflammatory innate responses [53]. 

Low vitamin D evident as a risk and predictor of COVID-19 severity

SARS-CoV-2 began to spread around the globe in late 2019, culminating in COVID-19, which has killed a large number of people. Thanks to the results of numerous research, the virus's genesis, toxicity, and transmission have all been explained. The pulmonary angiotensin-converting enzyme (ACE) 2 was introduced as the viral receptor for entering the cell, similar to the SARS coronavirus. According to an increasing amount of epidemiological and clinical data, vitamin D regulates lung damage via various pathways. The spread and severity of the illness have been related to several clinical signs as well as biological variables. Vitamin D, ACE, and the neutrophil to lymphocyte ratio (NLR) were assessed in COVID-19 patients and compared to a control group in the Mardani et al., 2020 research. In the patients' group, there were significant changes in vitamin D and ACE levels, as well as NLR. These variables have been shown to have an impact on the prognosis and severity of the illness [55]. 

In another single-center retrospective cohort analysis, vitamin D insufficiency was related to a greater risk of COVID-19. COVID-19+ individuals with sufficient vitamin D levels exhibited lower blood levels of D-dimer, the inflammatory marker CRP, fewer ground-glass opacities on chest CT images, and shorter hospital stays. According to the results, randomized trials should be performed to determine whether vitamin D levels influence COVID-19 risk. The research was not without flaws. Vitamin D insufficiency may be caused by several chronic illnesses or lifestyle choices, all of which are related to an increased COVID-19 risk. Furthermore, the research only looked at a tiny part of the data in the computerized database at Tokat State Hospital in Turkey [56].

Whilst levels of interleukin-6 (IL-6) are greater in cases of severe COVID-19 infection. This cytokine may play a far larger role in viral infection pathogenesis. Vitamin D is known to regulate IL-6, and its insufficiency is related to worse outcomes. Early COVID-19 mortality data from Italy and the United Kingdom were compared to previously published mean IL-6 levels from these nations, as well as the United States, to see if IL-6 levels before infection might predict the outcome. There was a very significant connection (r = 0.9883; p = 0.00025) between age-stratified death rates and IL-6 levels in previously published data on healthy individuals. To determine whether vitamin D might assist patients to decrease their IL-6 levels, a review of studies investigating the connection between these entities published in 2015 was performed. In eight of the eleven trials, vitamin D was shown to have a substantial decreasing impact on IL-6. Because IL-6 increases viral cell entrance and multiplication, it may be an excellent predictor of death before infection. This supports the use of preventive and therapeutic treatments, such as vitamin D administration, to lower IL-6 levels [57].

A meta-analysis of COVID-19 data from 532 hospitalized patients (189 on vitamin D supplementation and 343 on usual care/placebo) was conducted. Patients who took vitamin D supplements required less time in the ICU (p0.0001) than patients who did not take vitamin D supplements (OR=0.36; 95% CI: 0.210-0.626). It did, however, contain a lot of heterogeneity, which sensitivity analysis helped to decrease. In terms of mortality, vitamin D supplements are comparable to placebo treatment/standard care (OR=0.93; 95%CI: 0.413-2.113; p=0.87). There was no indication of publication bias in the studies. Subgroup analysis was not feasible due to the limited number of trials; thus, the dosage and duration-dependent impact of vitamin D could not be evaluated. According to the meta-analysis, more solid evidence from RCTs is required to support vitamin D's impact on mortality, despite the present results suggesting that it may have a role in decreasing COVID-19 severity in hospitalized patients [58].

## Conclusions

Suboptimal vitamin D is incriminated as a risk and outcome determinant for other diseases beyond abnormal bone health. Vitamin D is an essential nutrient that has the potential benefit of playing both therapeutic and preventive roles. Its use can be extended to tackle the two deadliest pandemics we are facing now, COVID-19 (communicable) and CVDs (non-communicable). Although more studies are emerging that highlight the epidemiologic, immunologic, and clinical relationship of vitamin D with various diseases, including COVID-19, further research warrants understanding its application and implication in public health intervention. 
